# Phenotypic Trade-Offs: Deciphering the Impact of Neurodiversity on Drug Development in Fragile X Syndrome

**DOI:** 10.3389/fpsyt.2021.730987

**Published:** 2021-10-18

**Authors:** Truong An Bui, Julie Shatto, Tania Cuppens, Arnaud Droit, François V. Bolduc

**Affiliations:** ^1^Department of Pediatrics, University of Alberta, Edmonton, AB, Canada; ^2^Centre de Recherche du CHU de Québec-Université Laval et Département de Médecine Moléculaire de l'Université Laval, Laval, QC, Canada; ^3^Department of Medical Genetics, University of Alberta, Edmonton, AB, Canada; ^4^Neuroscience and Mental Health Institute, University of Alberta, Edmonton, AB, Canada

**Keywords:** fragile X syndrome, trade-off, clinical trials, neurodevelopmental disorder, autism, intellectual disability, neurodiversity, phenotype-genotype correlation

## Abstract

Fragile X syndrome (FXS) is the most common single-gene cause of intellectual disability and autism spectrum disorder. Individuals with FXS present with a wide range of severity in multiple phenotypes including cognitive delay, behavioral challenges, sleep issues, epilepsy, and anxiety. These symptoms are also shared by many individuals with other neurodevelopmental disorders (NDDs). Since the discovery of the FXS gene, FMR1, FXS has been the focus of intense preclinical investigation and is placed at the forefront of clinical trials in the field of NDDs. So far, most studies have aimed to translate the rescue of specific phenotypes in animal models, for example, learning, or improving general cognitive or behavioral functioning in individuals with FXS. Trial design, selection of outcome measures, and interpretation of results of recent trials have shown limitations in this type of approach. We propose a new paradigm in which all phenotypes involved in individuals with FXS would be considered and, more importantly, the possible interactions between these phenotypes. This approach would be implemented both at the baseline, meaning when entering a trial or when studying a patient population, and also after the intervention when the study subjects have been exposed to the investigational product. This approach would allow us to further understand potential trade-offs underlying the varying effects of the treatment on different individuals in clinical trials, and to connect the results to individual genetic differences. To better understand the interplay between different phenotypes, we emphasize the need for preclinical studies to investigate various interrelated biological and behavioral outcomes when assessing a specific treatment. In this paper, we present how such a conceptual shift in preclinical design could shed new light on clinical trial results. Future clinical studies should take into account the rich neurodiversity of individuals with FXS specifically and NDDs in general, and incorporate the idea of trade-offs in their designs.

## Introduction

### Neurodevelopmental Disorders Are Characterized by Diverse and Complex Phenotypic Traits

The term neurodevelopmental disorders (NDDs) refers to a group of disorders marked by impairments of human functioning, including personal, social, academic, and occupational functions (1). Between 3 and 18% of the world's population is affected by NDDs (2–8). NDDs include a wide range of disorders such as global developmental delay (GDD), autism spectrum disorder (ASD), intellectual disability (ID), and attention-deficit hyperactivity disorder (ADHD) (9–14). These represent distinct diagnoses, and individuals can, therefore, present multiple diagnoses simultaneously. An individual who is diagnosed with a specific condition could present some phenotypes that are also found in another condition, as there are phenotypic overlappings between different disorders. Mental health disorders, in particular, are common comorbid conditions that affect many individuals with NDDs (7, 15). Genetic testing in individuals with NDDs has provided important insight into the molecular basis of this overlapping (16–18). Treatments of NDDs aim to provide solutions to the issues that individuals with NDDs commonly experience, such as limitations in daily living activities (19), barriers to participation in society (20), and lower quality of life compared to typically developing individuals (21).

Fragile X syndrome (FXS) represents the most common single-gene cause of ID and ASD (22) and has been at the forefront of targeted drug development (22, 23). Individuals with FXS display a wide range of symptoms to various degrees. Although many cardinal phenotypes have been observed in most individuals with FXS, such as ID, anxiety, stereotypic behavior, emotional lability, gaze avoidance, hyperactivity, and attention deficits, some variations across individuals have also been noted (24, 25). Since FXS is an X-linked disorder caused by a trinucleotide repeat expansion in the FMR1 gene, most females have both a functional and a silenced copy of the FMR1 gene, resulting in a less severe degree of cognitive impairment compared to males (22, 23). Nevertheless, females with FXS have been shown to present with enhanced anxiety relative to their counterparts (26, 27). FXS, as well as many other X-linked syndromes, underlines the importance of gene dosage and the complexity of gene interactions in explaining phenotypic manifestations, a theme that is relevant to most NDD genes.

### The Presence of Phenotypic Trade-Offs Has Been Previously Studied in NDDs

The concept of trade-offs, where an acquisition on one side could result in a loss on another, has been studied extensively in the field of business decisions (28). While this concept has not been investigated as much in medicine, its presence in clinical practice is evident as clinicians constantly have to rank the order of investigation procedures when choosing the initial treatment/intervention for a condition (29). Furthermore, there are multiple studies that show positive and negative correlations among genome-wide association study (GWAS) summary statistics in psychiatric disorders. Hübel et al. found that many psychiatric disorders have positive and negative correlations with body fat percentage (%) and fat-free mass. For instance, schizophrenia (SCZ), obsessive-compulsive disorder (OCD), and anorexia are negatively correlated with body fat % and fat-free mass, whereas a positive correlation is found between attention-deficit/hyperactivity disorder (ADHD) and these body composition factors (30). Tylee et al. also observed some significant correlations between immune-related disorders and several psychiatric disorders, such as Tourette syndrome, SCZ, bipolar disorder (BP), major depression (MD), and OCD (31). These findings align with our proposed model of trade-offs, with the elevation of one trait significantly affecting the expression of another seemingly unrelated trait. In fact, in a GWAS conducted in 2020, it was found that body mass index (BMI) and BP, SCZ, and MD have extensive polygenic overlappings and shared genetic loci, supporting their observed functional correlations.

**Figure 1:**
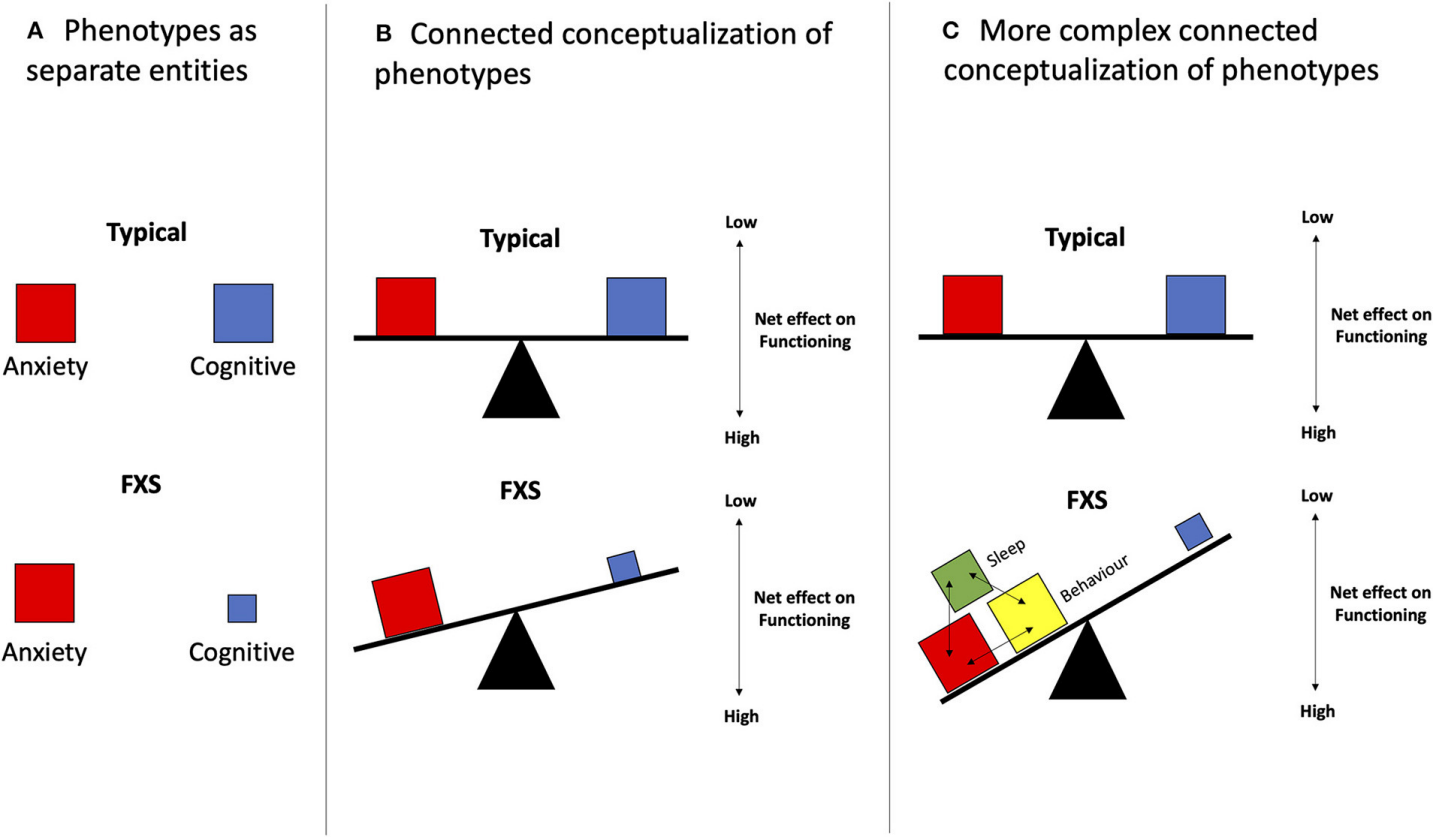
Phenotypic trade-offs can be conceptualized as variations between inter-related phenotypes. **(A)** Phenotypes are represented as separate entities. Viewing different phenotypes of a disorder as independent factors could impede the design and implementation phases of an intervention. **(B)** In its simplest form, 1:1 interactions of different phenotypes are seen. This can be visualized as the simpler seesaw model which suggests that two phenotypes, as illustrated in the red and blue boxes, have opposing relations, in which an increase in one phenotype (red) leads to a decrease in the other (blue). An example of that could be the balance between increased hyperarousal and decreased sleep. **(C)** As seen in FXS, different phenotypes interact and exacerbate the existing pathological effects on another phenotype. For instance, shown in this figure, is the additive effect of the red, yellow, and green boxes on the blue box. In addition, this second model depicts a tilting table atop three bases. These three bases reflect a more complicated model that takes into account the neuronal network, as well as multiple other underlying factors such as genetic makeups, epigenetics, and environmental factors, which could influence the presence of phenotypes (boxes), creating a more complex phenotypic output in the end.

Phenotypic trade-offs within a given group of individuals with the same diagnosis have been most extensively studied. For example, social skills are often impaired in individuals with ASD while other skills, such as visual processing, are improved (32). Similarly, the loss of empathy is usually associated with the gain in systematization skills (33, 34). These findings suggest that instead of being described as separate entities (Figure 1A), different characteristic phenotypes of a disorder could be represented as traits or behaviors that are negatively correlated with one another, like a seesaw, as illustrated in Figure 1B. Phenotypic trade-offs may offer various benefits to individuals. For instance, ASD patients have been observed to have more advanced visual-spatial skills compared to those with typical development (35–37). This trade-off could be tied to modifications in the neuronal network. Recent neuronal tract imaging using diffusion tensor imaging (DTI), which is a magnetic resonance imaging (MRI) technique, suggests that the trade-off might be related to an increased short-range circuit and decreased long-range connectivity (38–41) in ASD, and also abnormal connectivity in FXS (42, 43).

Beyond the seesaw model, a more realistic, yet also more complex representation of the phenotypic trade-offs seen in FXS and NDDs is shown in Figure 1C. In this case, some phenotypic traits may be positively correlated, in addition to having negative correlations. This model has been incorporated in many novel analytical methods using the multiple-symptom rating where several phenotypes need to move in the same direction without individually achieving statistical significance (44). When analyzing behavior in FXS, the increased expression of some phenotypic traits, for instance, challenging behaviors, was noted to be coupled with not only one, but also multiple other traits such as ASD-related symptoms including hyperarousal and social impairment (45). Other examples include the delay in toilet training being associated with sex (males), low intelligence quotient (IQ), behavioral problems, and delayed language capabilities (46). The factors that affect trade-offs also extend beyond features explainable by biological variations within neuronal networks and usually involve perplexing epigenetic and environmental elements. For instance, there was increased ASD in FXS patients with soy-based formula consumption and the opposite with breastmilk (47, 48). Other examples include high birth size (e.g., weight, length), which is associated with a higher risk of ASD but a lower risk of SCZ, and could be influenced by many prenatal factors. In contrast, low birth size has been connected to a lower risk of ASD and a higher risk of SCZ (49). Another example of the complex and rather mysterious biological interactions underlying NDDs is the “Protection-Against-Schizophrenia” (PaSZ) model. This model suggests that congenital blindness might serve as a “protection” mechanism against developing SCZ (50, 51), although this type of visual impairment has been shown to predispose to ASD features (52). These trade-offs highlight the intricate interactions between environment, epigenetics, and genetic features involved in phenotypic development (Figure 1C).

### Phenotypic Trade-Offs Are Considered When Choosing Clinical Pharmacological Interventions

When assessing the memory capacity in individuals with epilepsy, there have been some cases in which antiepileptic drugs successfully treat epilepsy-induced cognitive changes (53). This type of trade-off is the case for several different drugs and has been intensively investigated with topiramate. Topiramate was shown to prevent the shuffling of the glutamate receptor 1 (GluR1) subunit of the α-Amino-3-hydroxy-5-methyl-4-isoxazolepropionic acid (AMPA) receptor, which not only is associated with epileptogenesis but could also expose patients to learning or memory issues (54, 55). Considering the role of AMPA subunit shift in long-term potentiation (LTP), this result is not surprising, however, it is critical in confirming the connections between glutamate receptors, LTP, and epilepsy (56–58). Similarly, stimulant medications, such as methylphenidate formulations and amphetamine derivatives, are used for ADHD to improve attention deficits, yet, they could cause a subset of patients some sleep difficulties, leading to a lack of overall improvement (59, 60).

### Phenotypic Heterogeneity and Implications for Clinical Trials in FXS and NDDs

Although the clinical phenotypes associated with FXS have been well-established in the literature (61), the phenotypic trade-offs of FXS have yet to be extensively explored. Researchers and clinicians have reported some phenotypic variations among FXS patients, including those that are the molecular markers and core features of FXS, such as excessive protein synthesis (62–64). Considering that preclinical drugs are developed to rescue a specific phenotypic trait or only a limited number of traits, whereas the target population exhibits a high degree of phenotypic variations, it is likely that the preclinical results might not translate well. Thus, when developing therapeutic treatments for an NDD, it is imperative to consider not just the phenotypic presentation of the disorder, but also its genetic makeup and the biological interconnections underlying it. This issue extends beyond FXS, as many clinical NDD entities have now become defined by a genetic etiology instead of a specific symptom or a set of symptoms. This has been made possible with the growing accessibility and significant decrease in the cost of genetic testing. Genetic testing allows a wide range of genetically defined NDDs (65–70) to be more deeply understood through research programs (71), clinical testing (72, 73), and information sharing (74, 75). Consequently, the number of preclinical animal models and candidate drugs has proliferated.

In this paper, we discuss how considering single or multiple phenotypes and their interactions (Figure 1), which we conceptualize as trade-offs, could provide some insights into the most recent trials and could generate a new framework for preclinical, clinical studies, and patient registries.

## Hypothesis

We posit that in each individual with FXS, the multiple phenotypic traits may interact with one another through complex and multidirectional networks. While an intervention may improve one target phenotype (TP), it could have a negative or absent effect for another unexpected phenotype (UP), leading to either a null net gain or a worse outcome for the individual. The UP could mask or occlude the improvement in the TP. This multiplex understanding of phenotypes is important as it allows one to uncover the gain in a TP after correcting the UP.

## Observations

There are multiple potential explanations for the failure to achieve desirable impacts from candidate drugs in a clinical trial for FXS, including challenges in study design, outcome measures selection, finances, recruitment, and participant retention (76, 77). By discussing the results of recent clinical trials in FXS, we identify a possible new explanation for the loss of net gain from their pharmacological treatments. We look at substantiated preclinical studies to assess the possibility that UP might have exerted an epistatic effect on the TP, which could mask the potential benefit from the intervention.

### Phenotypic Trade-Offs and Recent Clinical Trials With Individuals With FXS

Several pharmacological targets have been considered for the treatment of cognitive and behavioral phenotypes experienced by individuals with FXS. Here, we focus on two targets subjected to clinical trials in the last 5 years, the metabotropic glutamate receptor (mGluR) and cyclic adenosine monophosphate (cAMP) signaling pathways. A number of studies have shown that group II and III mGluRs are negatively correlated with cAMP. In the cerebellar granule cell neurons (78), striatal neurons (79), hippocampal neurons (80), and cerebellar astrocytes (81) of rats, the activation of mGluR3 receptors is followed by decreased cAMP level. mGluR agonists have been shown to inhibit adenylyl cyclase and reduce neuronal death, which is modulated by inhibiting cAMP (82). Although findings on the effects of mGluR agonists and antagonists on the cAMP signaling pathway are extensive, further studies are needed to illustrate how gene expressions in these two pathways are connected. Some directions for these studies have been proposed. For instance, if the administration of mGluR inhibitors was shown to rescue cAMP deficits, this would indicate that cAMP defects are downstream of excessive mGluR activity (83).

Inhibition of mGluR was shown first in FMR1 knock-out (KO) mouse hippocampal brain slices (84, 85) and then, in the FXS fly model (86) to rescue the excessive long-term depression (LTD) in the hippocampus, a key structure for learning (87–90). These promising results were translated into two large industry-led clinical trials (91, 92). Benefits from the trials were highly expected as mGluR5 inhibitors were also shown to have the anxiolytic effect in wild-type (WT) mice and rats (93, 94). Unfortunately, the results of these trials failed to show an improvement in their behavior measure (91, 92). Nevertheless, the trials still revealed many new insights. FXS patients on mGluR inhibitors presented a trend towards a higher degree of anxiety (−8.63 ± 1.55), measured by mean ± standard error of the mean (SEM), according to the Anxiety Depression Assessment Measure Scale (ADAMS), than the placebo group (−10.63 ± 1.49) (92) (Table 1). In addition, measures of insomnia and agitation were also showing a trend toward more severe issues with higher doses of mGluR inhibitors (Table 1). While the trials failed to deliver desirable outcomes, their results could be explained with an analysis of phenotypic trade-offs, particularly by considering the interrelationships between cognitive skills (TP) and anxiety (UP). This is possible as increased anxiety has been shown in the literature to negatively affect cognitive performance in general (96, 97) and in FXS in particular (98).

**Table 1:** Summary of clinical trials' results investigating the behavioral effects of mGluR (AFQ056-Mavoglurant, Basimglurant) and cAMP (BPN14770) signaling inhibitors.

	**Hagerman et al**. **(****91****)**				**Youssef et al**. **(****92****)**			**Berry-Kravis et al**. **(****95****)**	
	**mGluR signaling inhibitor**				**mGluR signaling inhibitor**			**cAMP signaling inhibitor**	
	**AFQ056 25 mg**	**AFQ056 50 mg**	**AFQ056 75 mg**	**AFQ056 100 mg**	**Placebo**	**Basimglurant** **0.5 mg**	**Basimglurant** **1.5 mg**	**Placebo**	**BPN1477025 mg**
**Mean (SEM)**									
ADAMS	—	—	—	—	−10.63 (1.49)	−6.20 (1.69)	−8.63 (1.55)		
Anxiety^a^								−0.79	−1.41
ABC	—	—	—	—	−16.26 (2.81)	−10.46 (3.11)	−11.53 (2.91)		
Irritability								0.91	−0.71
Hyperactivity								1.19	−0.12
CGI-S	—	—	—	—	−0.26 (0.07)	−0.26 (0.08)	−0.27 (0.1)		
CGI-I	—	—	—	—	3.06 (0.11)	3.47 (0.12)	3.39 (0.16)		
SRS T-score	—	—	—	—	−8.25 (1.44)	−3.65 (1.58)	−4.65 (1.48)		
RBANS	—	—	—	—	0.69 (1.52)	1.37 (1.67)	0.87 (1.57)		
VAS	—	—	—	—	−20.35 (4.03)	−12.45 (3.34)	−16.11 (4.20)		
Anxiety^b^								5.96	9.07
Irritability^c^								3.86	17.91
VABS-II	—	—	—	—	3.93 (2.57)	1.70 (2.80)	2.71 (2.50)		
***N*** **(%)**									
Aggression	3 (2.5)^†^	3 (2.5)^†^	4 (3.4)^†^	12 (11.1)^†^	1 (1.6)	5 (8.6)	5 (8.1)	—	—
	6 (4.1)^††^	8 (5.4)^††^	6 (4.3)^††^	12 (8.9)^††^					
Insomnia	4 (3.4)^†^	10 (8.5)^†^	5 (4.3)^†^	12 (11.1)^†^	1 (1.6)	0 (0)	5 (8.1)	0 (0)	1 (3.3)
	4 (2.7)^††^	3 (2.0)^††^	7 (5.0)^††^	12 (8.9)^††^					
Anxiety	3 (2.5)^†^	1 (0.8)^†^	0 (0)^†^	11 (10.2)^†^	1 (1.6)	2 (3.4)	6 (9.7)	—	—
	1 (0.7)^††^	3 (2.0)^††^	3 (2.1)^††^	10 (7.4)^††^					
Irritability	3 (2.5)^†^	1 (0.8)^†^	3 (2.6)^†^	9 (8.3)^†^	1 (1.6)	3 (5.2)	3 (4.8)	—	—
	0 (0)^††^	7 (4.7)^††^	4 (2.8)^††^	6 (4.4)^††^					
Agitation	0 (0)^††^	1 (0.7)^††^	3 (2.1)^††^	12 (8.9)^††^	2 (3.2)	0 (0)	4 (6.5)	—	—

*A decrease in ADAMS, Anxiety, Depression and Mood Scale; ABC, Aberrant Behavior Checklist; CGI-S, Clinical Global Impression–Severity scale; CGI-I, Clinical Global Impression-Improvement scale; SRS, Social Responsiveness Scale, and VAS scores indicates improvement. An increase in the RBANS, Repeatable Battery for Neuropsychological Status and VABS-II, Vineland Adaptive Behavior Scales II score indicates improvement. OCD, Obsessive-Compulsive Disorder. VAS, Visual Analog Scale. cAMP, cyclic adenosine monophosphate; mGluR, metabotropic glutamate receptor; SEM, standard error of the mean. If not specified, data was generated from participants of all age groups*.

† *Adolescents (12–17 years old)*.

†† *Adults (18–50 years old)*.

a *General anxiety*.

b *Anxiety/Irritability*.

c *Irritability/Language*.

*The clinical effects of the placebo group in Hagerman et al. (91) were not reported to the same extent as the treatment groups, thus, were not included in this table*.

To better understand how mGluR inhibitor could lead to enhanced anxiety in FXS, contrasting with its anxiolytic role in typical individuals, we sought to explore further quantitative measures of anxiety in FXS, specifically pupillary size measurement. Individuals with a high degree of anxiety often experience pupillary constriction and people with ASD have larger tonic pupil sizes compared to the neurotypical group (99–101). FXS individuals have been observed to present with significantly bigger pupils than typically developing (TD) individuals in response to pictures of human faces containing emotions (102). What was surprising is that treatment with mGluR inhibitor led to increased dilatation in response to neutral faces (103).

Amygdala is a key component of pupillary dilation. This suggests that amygdala might play a role in the precedent networks of anxiety, glutamate receptor signaling cascade, emotion recognition (104), pupillary dilation (105), and FXS. Although hypothetical at this time, it will be worth investigating further, as individuals with FXS have been shown to have abnormal amygdala connectivity (105, 106), and FMR1 KO mice have been also documented to exhibit amygdala-related behavioral deficits (107, 108). While these results are suggestive, it is also important to note that pupillary dilation can be seen with cognitive processing (109).

Another insight into the enhanced anxiety comes from preclinical studies showing a divergence between mGluR signaling mechanisms in amygdala vs. cortex and hippocampus. While the excessive activity of LTD was observed in the hippocampus, defects in LTP have been observed in amygdala of FMR1 KO mice (89). mGluR inhibitors were shown to rescue presynaptic but not postsynaptic defects (89), which is the opposite of what was seen in the hippocampus of the same group. This evidence suggests an interplay between the LTP and LTD, and hippocampus and amygdala, in relation to anxiety and memory. Hence, one could hypothesize the increase in anxiety following the mGluR treatment could occlude the improvement in memory.

Together, this evidence brings forth the importance of designing a comprehensive map of phenotypic interactions based on the diverse representations of the patient population.

### Differences in Molecular Signaling Pathways May Lead to Variable Trade-Offs Between Phenotypes

Next, we wondered if improvement in memory would always be poised to lead to enhanced anxiety leading to potential no net gain. Fortunately, a study recently published targeting another candidate pathway for FXS showed that trade-offs may be pathway-specific.

The intracellular second messenger cAMP and its downstream signaling cascades have been studied extensively with regard to learning in various animal models (14, 110–112). Berry-Kravis was among the first to show how cAMP induction is defective in FXS (113, 114). This result was replicated again in other animal models (83, 113–116), prompting a clinical trial testing the effects of PDE inhibitors in humans (95). The trial implemented a cross-over design, included a total of 30 participants, and showed some promising effects of the treatment on communication, language abilities as measured by the National Institutes of Health Toolbox Cognitive Battery (NIH-TCB) assessment tools, and other daily functioning (95). The study showed a trend of improvement in anxiety, as the treatment group scored a mean of −1.41 on the ADAMS scale compared to the placebo group (−0.79), although the statistical result was not significant (Table 1).

From these studies, we attempt to explain how phosphodiesterase (PDE) inhibitors could possibly lead to cognitive improvement without elevating anxiety as was seen in the case of mGluR inhibitors (Table 1). The answer might lie in the fundamental difference between the cAMP pathway and the mGluR pathway, which could also shed light on selecting new drug candidates developed to address both TP and UP. It is important to reiterate that the PDE trial (95) only included a small number of participants and their results need to be replicated in phase 3 of trial.

As for mGluR inhibitors, PDE inhibitors have been linked to having anxiolytic effects, influencing neurogenesis, and mitigating the effects of corticosteroids (117–121). Evidently, the study of Beer et al. (122) has shown how PDE inhibitors could reduce anxiety by elevating cAMP levels. However, this is contradictory to some studies that showed increased cAMP levels recorded following stress exposure in rats (123, 124). Data from studies in mice also showed that decreased PKA, which is an effector molecule downstream of the cAMP regulatory unit (PKA reg), was associated with increased anxiety and higher PKA activity in the basolateral and central amygdala, as well as in the ventromedial hypothalamus (125, 126). Consistently, increased PKA level from early life stress has been observed (117). Therefore, it remains unclear how PDE inhibitors could exert anxiolytic effects via the regulation of cAMP signaling. Another approach toward finding the connections between PDE inhibitors, cognitive performance, and anxiety is by looking at the activation of cAMP-response element binding protein (CREB) via the cAMP signaling pathway. This activation may lead to increased levels of neuropeptide Y (123), which is expressed in the amygdala.

There is a likelihood that FXS patients who were treated with PDE inhibitors would not experience the detrimental consequences of elevated activities of glutaminergic and cAMP signaling in the amygdala. As Kelley et al. demonstrated, the levels of cAMP were low in the amygdala in FMR1 KO mice (127), which suggests that PDE inhibitors might compensate for this defect in FXS patients.

## Discussion

In this paper, we discuss some important factors to consider when assessing the molecular basis and neurological circuitry of phenotypic trade-offs in NDDs, and FXS in particular. It is clear that more studies need to be done to test the hypothesis that we propose, which outlines that pharmacological interventions must account for potential interacting phenotypic trade-offs. This framework could provide a more personalized and precise approach for future clinical trials.

### Translational Caveats Impact Accurate Assessments of Phenotypic Trade-Offs

**Figure 2:**
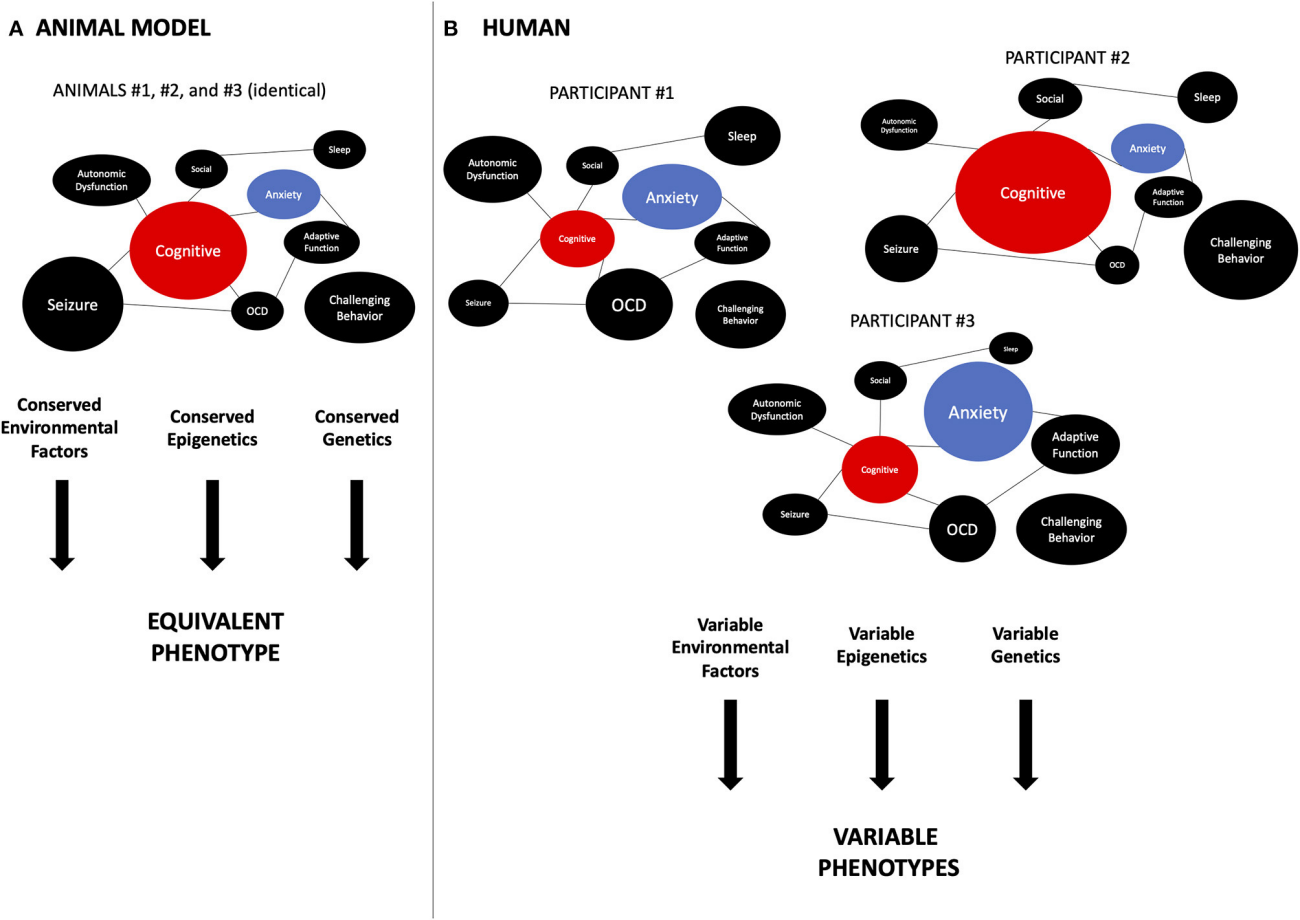
Translational aspects related to phenotypic trade-offs. An important difference between preclinical studies in animal models and humans is the degree of inter-individual variability in phenotypes. **(A)** In animal models, especially the inbred models used in the laboratory, genetic background, and environmental conditions are controlled closely. This leads to very homogeneous phenotypes, as each circle represents a phenotype seen in FXS and the size of the circle is representing the severity of the phenotype. This allows behavioral researchers to obtain more significant results. As the weight of each phenotype is conserved between individual animals, so is the interaction between phenotypes. For instance, the cognition (TP) (red) is always facing the same weight of anxiety (UP) (blue). Thus, a drug aimed at the TP will always interact with the same amount of potential effect of UP. On the other hand, **(B)** in human phenotypes, both cognition and anxiety vary significantly between individuals, as the size of each circle represents the magnitude of the severity of the phenotype. Multiple reasons could explain this, including genetic background, variable genetic lesion in the target gene, environmental, epigenetic, demographic, etc. The variations in TP and UP can influence the impact of trade-offs. For instance, in participant #1, the impact of anxiety is much bigger than in participant #2. So a drug targeting cognition (TP) positively but also enhancing anxiety (UP) will seem to have a great effect in individual #2 but be detrimental to participant #1 or #3. In a more realistic scenario, there are more than two phenotypes interacting, hence, the relative balance of those phenotypes is often unknown in clinical trials, making it difficult to analyze and predict the outcomes of the intervention. FXS, fragile X syndrome; TP, target phenotype; UP, unexpected phenotype; OCD, obsessive-compulsive disorder.

As shown by the above analysis of recent FXS clinical trials, translating research results obtained from animal models into clinical settings, which involve testing drugs to target multiple cognitive-related functions in humans, can be difficult (128, 129). To ensure a more successful translation from animal research into human clinical trials, a better understanding of trade-offs is needed. However, studying trade-offs in humans may pose many challenges due to the fact that the human model is more complex and sophisticated. Furthermore, unlike animal models, which have homogeneous phenotypes (Figure 2A), humans present a high degree of phenotypic variation (Figure 2B), even within a given genetic diagnosis (130). These interconnected traits could be traced back to variations at both molecular level and tissue level. Hence, when developing pharmacological treatments, it is crucial to not only assess the phenotypes expected to be affected by the drug, but also to understand how the genetic and molecular makeups of an individual might differ from the average. By building a schema of interactive phenotypic, genotypic, and molecular mechanisms, we ensure a more efficient development of the treatment and a better outcome for patients. Objective measures that involve multi-domain tests and performance-based tasks with regard to testing cognitive functions, could allow us to fathom the trade-off effects of the treatment on intra- and inter-individual bases.

### Biological Factors Can Influence Phenotypic Trade-Offs

**Figure 3:**
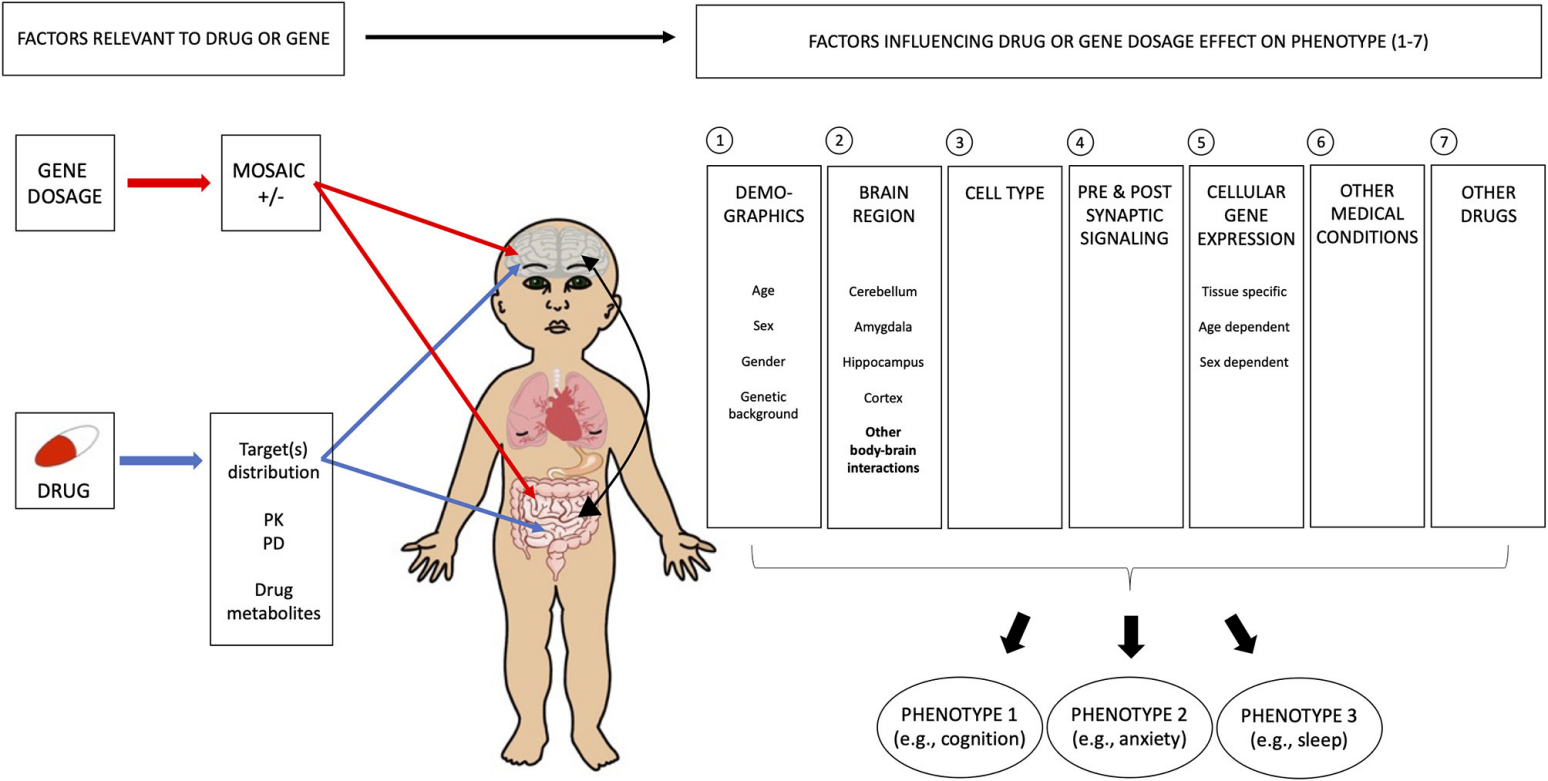
Multiple biological factors contribute to the complexity of phenotypic trade-offs. Several general variables need to be considered in understanding the biological basis of phenotypic trade-offs, as seen with drug treatment. In the case of FXS, it is important to include the level of FXS protein, FMRP, as this has an effect on IQ. Mosaicism also influences the phenotypic severity of IQ and potentially other phenotypes. However, this is not well-understood. In addition, drug and gene-specific variables need to be taken into account, such as (1) brain region, (2) cell type, (3) synaptic location (pre vs. postsynaptic signaling), and (4) gene expression. For (1) brain region, we imply that the effects of gene mutation and drugs should be understood for all disorder-relevant circuits. For instance, patients with disorders could present a cognitive deficit as well as an excessive anxiety, such that both clinical and preclinical data for these two phenotypes should be assessed. The potential trade-offs between these two phenotypes should also be evaluated. (2) Cell type needs to be considered in terms of the patterns of gene expression and effects of the drug. For instance, FXS has a role in both excitatory and inhibitory neurons, and a drug affecting one and not the other may worsen the patient's condition where homeostasis should be conserved. Similarly, the imbalance between glia and neurons, and more recently, between microglia and inflammation could lead to unexpected effects. Changes in cells from specific cortical layers may affect network properties of one phenotype more than another. These could be more systematically investigated in the preclinical field to provide further insights. (3) Synaptic location plays an important role in the net effect of an intervention. As seen in the case of mGluR, it is important to understand the impact of regional variations in this distribution. Finally, variations in patterns of (4) gene expression within the brain, and in the body, probably play a major role in explaining changes in behavioral outputs. For instance, demographic factors, such as sex and age, have important impacts on the ability of a drug to affect a behavioral phenotype. What is more complex is that although developmental windows are altered in neurodevelopmental disorders, the window for each phenotype may not be delayed in the same way, leading to variable inter-phenotype relation overdevelopment, as well as the closure of some windows before others, which all affect observed trade-offs between phenotypes. FXS, fragile X syndrome; FMRP, fragile X mental retardation protein; IQ, intelligence quotient; PK, pharmacokinetics; PD, pharmacodynamics.

As indicated by differences in the trade-offs between treatments targeting mGluR and cAMP, variations in biology may significantly influence developments of such trade-offs. We discuss here some of the factors to consider, and we suggest investigating them both pre-clinically and clinically. The elements that link the molecular actions of a pharmacological agent to the corresponding behavioral outcomes are numerous, and pertains in part to the pharmacological agent, the genetic disorder, and its effect on the intrinsic features of human biology (Figure 3). Gene dosage refers to the number of copies a specific gene can have in the genome. Extreme changes are observed when comparing duplication and deletion for the same chromosomal region, which show diametrically opposite phenotypes. Individuals with chromosome 1q21 duplications present increased prevalence of autism and macrocephaly, whereas 1q21 deletion is associated with increased SCZ rate and microcephaly (131–133). Similarly, while 16p11.2 duplication and deletion share many common features, they also have opposing phenotypes (134). Individuals with deletion of chromosome 16p11.2 show significantly higher frequencies of functional motor abnormalities (e.g., oro-motor articulation and agility) and hyporeflexia, as well as macrocephaly (134–137) and bigger cerebellar volume (138), compared to typical individuals. On the other hand, individuals with duplications of 16p11.2 present hyperreflexia, tremor, microcephaly (134–137), and decreased cerebellar volume (138). Milder changes in gene dosage can also have an important impact on phenotypes. Indeed, the level of FXS protein, FMRP, is correlated with IQ (139, 140), but the relation with other phenotypes is not as clear. In addition, mosaicism will influence phenotypic severity for IQ and potentially other phenotypes, but this is not well-understood (141, 142). Gene dosage has variable effects on the efficacy of a drug and the expression levels of different phenotypic traits in an individual. This interplay between gene dosage and the drug, as well as their collective effects on the peripheral, autonomic, and enteric nervous systems, should be highly considered in pharmaceutical designs, as shown recently in SCZ (143, 144).

There are many other reasons why a pharmaceutical agent might cause unforeseen negative effects on the UP. This could be due to the variations in signaling pathways between brain regions as the result of differential gene expression patterns or temporal gene expression changes. The agent might also act differently at the presynaptic and postsynaptic levels, as seen in the case of opposing effects of mGluR signaling in amygdala and hippocampus. Examining the interactive roles of multiple organs might also be essential for us to gain deeper understanding of the unforeseen trade-offs indicated by the emergent role of the gut microbiome on neurological disorders, such as depression and autism (145–149). Even within a given brain region, there are still notable differences in gene expression across cell types, thus, differences in signaling pathways, as shown with ASD (150). Sex differences might also offset the phenotypic balance and cause a variety of reactions in response to a particular drug (151). Lastly, differences in age could induce significant variations in not only gene expression but also morphological features, as shown in previous studies conducted in FXS mouse models of different developmental ages with opposing dendritic spine structures (152).

### Genetic Backgrounds Complicate the Understanding of Phenotypic Trade-Offs

The challenges in understanding phenotypic variations (Figure 2) and phenotypic trade-offs can also be attributed in part to the complex nature of the human genetic background and epigenetic makeup. A better knowledge of genetic backgrounds is needed to understand phenotypic trade-offs, as it has been shown to modulate the expression of the behavioral phenotype in both fly (153–155) and mouse (156, 157) models. In FMR1 KO mice, systematic comparative analysis between strains revealed important differences between strains from the masking of some phenotypes to the exacerbation of others (156–158). However, this has been seen as well in other neurological disorders such as Huntington's disease (HD) (159). The genetic background makeup may contribute to the phenotypes in a manner not directly related to the target gene mutation. This has been labeled as the two-hit hypothesis (160). This implies that a better understanding of the genetic background and its targeting by the drug may shed light on complex trade-offs.

### Understanding Phenotypic Trade-Offs Through Evolutionary Perspectives

Genes may also serve different purposes at different time points, a concept known as pleiotropy. Similar to trade-offs, antagonistic pleiotropy posits that some trade-offs may exist between phenotypes over time by enhancing a certain gene. Recently, a well-known gene involved in neuronal activity, learning, and memory, calcium/calmodulin-dependent protein kinase II (CaMKII), was shown to be beneficial to young flies and mice, but to lead to a higher susceptibility to aging-related diseases in older animals due to its interaction with reactive oxygen species (161). This raises the need to extend our understanding of trade-offs outside of brain-related functions. For instance, cancer and Alzheimer's disease (AD) may also be in a trade-off position. There are 286 genes that have been found to overlap between AD and cancer, which links more than 60% of AD-associated genes with cancer (162). One of these genes is PIN1, which codes for a protein that regulates cell proliferation and survival. The genetic variant −842G>C in the promoter region of PIN1 gene is associated with increased risks of cancer and decreased risks of AD (163, 164). Several authors have proposed that the cause of one disease (e.g., cancer) might serve as the protective mechanism for others such as AD, HD, or Parkinson's disease (PD) (165–167). On the other hand, for PD and cancer, a meta-analysis of 29 studies has shown that of 107,598 patients with PD diagnosis, there was a 27% decreased risk of all types of cancers and a 31% reduced risk of cancer excluding melanoma and other skin cancers (168). Similar results have been obtained in other studies (169).

## A New Approach

We propose designing and testing interventions in FXS and other NDDs, with the hypothesized notion that potential trade-offs between phenotypes may exist at the baseline in patients and impact their TP readouts. For instance, an individual may have low memory performance because of a deficit in the memory function or of exaggerated anxiety masking memory function. Moreover, we suggest taking trade-offs into account during both the design and interpretation phases of an intervention, regardless of whether it is pharmacological or not. Trade-offs could dictate the net effect of the intervention on a phenotype and could be better understood with the analysis of the TP, for example, memory, and also the UP. These interacting phenotypes could greatly affect the penetrance of the effect of the intervention. We propose that changes in the way that both preclinical and clinical research are conducted would be important in obtaining critical quantitative data that are needed to test this hypothesis.

### Preclinical Perspective

From a molecular point of view, it will be important to better define regional (e.g., brain regions, cell types, pre vs. postsynaptic and extra-nervous system location), sex, developmental-related differences in pathway signaling (e.g., metabolomics and proteomics), and gene expression profiles of the individuals. This will help anticipate potential antagonistic or synergistic effects of a potential intervention on certain phenotypes. As we discussed in cases of mGluR and cAMP pathways, a given signaling pathway could differ between two different brain regions such as amygdala and hippocampus (107). Most laboratories will usually focus on one brain region related to a behavior of interest. It would be important to consider assessing the effect on other brain regions, which may show a negative impact on the TP, prior to clinical trials. Moreover, it may be important to know whether a drug also has a positive effect, or improves, a phenotype (UP) that is antagonistic to the TP. For instance, if a drug not only improves memory (TP) but also reduces anxiety (UP), then it may create even more benefits, as increased anxiety would impair learning and memory at the baseline in an individual with FXS. This may “unmask” some existing potential beneficial phenotypes that were not expressed because of the antagonistic phenotype.

Similarly, from a behavioral or phenotypic point of view, it will be important to assess a variety of disorder-relevant phenotypes in parallel for each given intervention. For instance, we have shown that PDE inhibitors may require a different dosing or even a different signaling pathway when targeting learning vs. anxiety-like behavior (170). In addition, it is common in the clinical practice of medicine to note that a drug may improve attention but have a negative effect on sleep (107, 171).

### Clinical Perspective

We propose to consider a more systematic set of outcome measures (e.g., phenotypes) in all clinical trials in order to be able to compare drug trial to drug trial based on their impacts on behavior. Having discussed this with several parents and investigators in the field of FXS, we propose that it would be important to (1) establish a consensus list of TPs that would benefit from interventions; (2) identify quantitative and objective measures of those phenotypes; (3) consider analyzing phenotype interactions at baseline and after intervention at both intra-individual level and inter-individual level (considering the importance of neuro-diversity). This would allow us to further understand why some individuals may respond differently than others. It would also be key in concatenating the information from one trial with another. This will be challenging with the current trial setup with small participant number and we hope our proposed approach will assist in the development of more multicentric coordinated trials.

From a molecular perspective, it remains difficult to assess the regional effect of a drug and its correlation with phenotypic outcomes. Assessing local changes in brain activity using electroencephalography (EEG) or magnetoencephalography (MEG) or functional magnetic resonance imaging (fMRI), both at baseline (e.g., before starting administering the drug) and after intervention, could provide significant insights into that. In addition, it will be important to consider the presence of mosaicism in the brain which could impact the drug-phenotype interactions (172, 173), although, at this time, its assessment is not possible within the brain in humans.

Finally, we would like to state that while we have put forward some general ideas here, we suggest that more extensive discussions will be required, which could encourage the creation of an overarching consortium including parents and scientists conducting both preclinical and clinical studies on interventions in NDDs aimed at developing a broad consensus. We also propose taking a more quantitative, data-driven approach, such as applying machine learning (ML) and other artificial intelligence (AI) methods in the studies of trade-offs.

## Data Availability Statement

The original contributions presented in the study are included in the article, further inquiries can be directed to the corresponding authors.

## Author Contributions

TAB assisted with literature review and co-wrote the paper. JS assisted with figure preparation. TC and AD assisted with research concept. FB developed the hypothesis, performed the literature review, co-wrote the paper and developed the figure concepts. All authors contributed to the article and approved the submitted version.

## Funding

This work was funded by CIHR and NSERC.

## Conflict of Interest

The authors declare that the research was conducted in the absence of any commercial or financial relationships that could be construed as a potential conflict of interest.

## Publisher's Note

All claims expressed in this article are solely those of the authors and do not necessarily represent those of their affiliated organizations, or those of the publisher, the editors and the reviewers. Any product that may be evaluated in this article, or claim that may be made by its manufacturer, is not guaranteed or endorsed by the publisher.
